# Detection of Nuclear Protein Profile Changes by Human Metapneumovirus M2-2 Protein Using Quantitative Differential Proteomics

**DOI:** 10.3390/vaccines5040045

**Published:** 2017-12-03

**Authors:** Yuping Ren, Eunjin Choi, Ke Zhang, Yu Chen, Sha Ye, Xiaoling Deng, Kangling Zhang, Xiaoyong Bao

**Affiliations:** 1 Department of Pediatrics, University of Texas Medical Branch, Galveston, TX 77555, USA; ypren@tjh.tjmu.edu.cn (Y.R.); euchoi@utmb.edu (E.C.); yuchen@tjh.tjmu.edu.cn (Y.C.); cindy.ye.dbm@gmail.com (S.Y.); xideng@utmb.edu (X.D.); 2 Department of Plastic Surgery, TongJi Hospital, TongJi Medical College, Huazhong University of Science and Technology, Wuhan 430073, China; 3 Department of Biochemistry, Baylor University, Waco, TX 76706, USA; Ken_zhang@baylor.edu; 4 Department of Pediatrics, TongJi Hospital, TongJi Medical College, Huazhong University of Science and Technology, Wuhan 430073, China; 5 Department of Gynecologic Oncology Ward V, Hunan Cancer Hospital, Xiangya School of Medicine, Central South University, Changsha 410008, China; 6 Department of Pharmacology and Toxicology, University of Texas Medical Branch, Galveston, TX 77555, USA; kazhang@UTMB.EDU; 7 Sealy Center for Molecular Medicine, University of Texas Medical Branch, Galveston, TX 77555, USA; 8 The Institute of Translational Science, University of Texas Medical Branch, Galveston, TX 77555, USA; 9 The Institute for Human Infections & Immunity, University of Texas Medical Branch, Galveston, TX 77555, USA

**Keywords:** hMPV, M2-2 motif, proteomics

## Abstract

Human metapneumovirus (hMPV) is a leading cause of lower respiratory infection in pediatric populations globally. This study examined proteomic profile changes in A549 cells infected with hMPV and two attenuated mutants with deleted PDZ domain-binding motif(s) in the M2-2 protein. These motifs are involved in the interruption of antiviral signaling, namely the interaction between the TNF receptor associated factor (TRAF) and mitochondrial antiviral-signaling (MAVS) proteins. The aim of this study was to provide insight into the overall and novel impact of M2-2 motifs on cellular responses via an unbiased comparison. Tandem mass tagging, stable isotope labeling, and high-resolution mass spectrometry were used for quantitative proteomic analysis. Using quantitative proteomics and Venn analysis, 1248 common proteins were detected in all infected samples of both technical sets. Hierarchical clustering of the differentiated proteome displayed distinct proteomic signatures that were controlled by the motif(s). Bioinformatics and experimental analysis confirmed the differentiated proteomes, revealed novel cellular biological events, and implicated key pathways controlled by hMPV M2-2 PDZ domain-binding motif(s). This provides further insight for evaluating M2-2 mutants as potent vaccine candidates.

## 1. Introduction

Human metapneumovirus (hMPV), a negative-sense single-stranded RNA virus, belongs to the *Pneumoviridae* family, the same virus family that causes mumps and parainfluenza [1,2,3]. Since its identification in 2001, hMPV has been isolated from individuals of all ages across the world who have acute respiratory tract infections [4]. However, there are currently no effective vaccines or specific therapeutic reagents available for individuals with hMPV.

Efforts are being made to develop vaccines against hMPV. However, hMPV challenge with formalin-inactivated hMPV enhances pulmonary disease and Th2 response, suggesting that inactivated hMPV may not be a suitable vaccine candidate [5,6]. Several viral protein-based vaccine candidates have been developed recently, with some inducing a strong humoral immune response against both homologous and heterologous strains. However, such response diminishes rapidly over time, which might result from the lack of other viral protein(s) contributing to the immunogenicity and immune balance [7]. The other important group of vaccine candidates is recombinant live attenuated hMPV. Recently, a wild-type recombinant hMPV was approved as a suitable parent virus for the development of live attenuated hMPV vaccine candidates in experimental human infection trials [8], providing a promising vaccine study direction for hMPV. We and others have developed several attenuated recombinant strains of hMPV by gene deletion or mutations [9,10,11,12,13]. Compared to other vaccines, live attenuated vaccines offer several advantages for the immunization of infants and young children, including no vaccine-associated enhanced viral disease, the induction of both humoral and mucosal immunity, intranasal vaccine delivery, and viral replication in the upper respiratory tract of young infants despite the presence of passively acquired maternally-derived respiratory syncytial virus (RSV) neutralizing antibodies [14].

**Figure 1:**
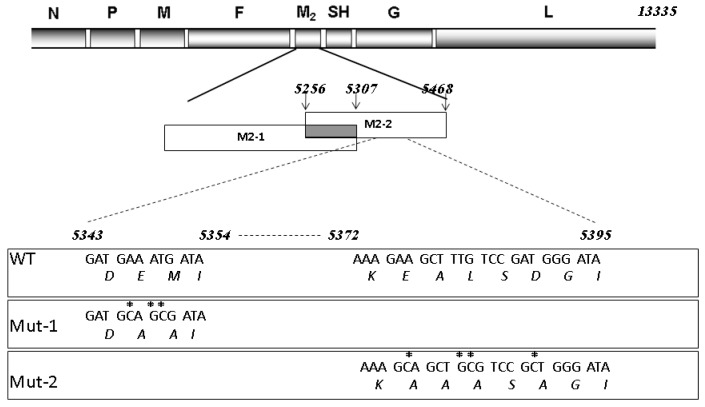
Map of human metapneumovirus (hMPV) antigenome and the mutations introduced to generate the M2-2 mutants. The overlapped open reading frame of M2-1 and M2-2 are shown as a gray rectangle with oligonucleotide sites given above. M2-2 amino acids labeled with stars were mutated to alanine to abolish interested PDZ binding motifs.

There are nine viral proteins associated with hMPV. Several laboratories including ours have demonstrated the functions of some viral proteins, among which phosphoprotein P, glycoprotein G, small hydrophobic SH, and M2-2 proteins are important for the regulation of host innate immunity [9,12,13,15,16,17,18]. In terms of M2-2, we have recently shown that it contributes to the immune evasion of infected human dendritic cells and airway epithelial cells by targeting myeloid differentiation primary response gene 88 (MyD88) and the mitochondrial antiviral-signaling (MAVS) protein, respectively [12,18]. In airway epithelial cells, two putative PDZ-binding motifs, 29-DEMI-32 and 39-KEALSDGI-46, are responsible for M2-2-mediated immune evasion. The mutations in the PDZ motifs enhance the interaction between MAVS and TRAFs, which subsequently induces a stronger host innate immune response to hMPV [13]. Importantly, these mutants do not alert the expression ratio of F and G proteins of viral particles (Supplementary Materials Figure S1) and thereby provide reliable viral sources for the immunogenicity and well-balanced T cell responses; surface proteins of many respiratory viruses are critical in mediating virus entry, immunogenicity, and the Th1–Th2 response balance [19,20,21]. In addition, the M2-2 mutants still carry the cytotoxic T lymphocyte epitope [22] and are, therefore, less likely to affect the cytotoxic T cell responses. Although recombinant viruses with mutations in the 29-DEMI-32 (Mut-1) or 39-KEALSDGI-46 (Mut-2) motifs, compared to wild-type (WT) hMPV, are attenuated and are, therefore, promising vaccine candidates [10], comprehensive studies on the impact of motifs on host responses are still needed for the mutants’ translational application. In Figure 1, the antigenome of WT hMPV and how the oligonucleotide mutations were introduced to generate Mut-1 and Mut-2 are illustrated.

Quantitative differential proteomic analysis of experimental and clinical samples using isobaric tags for relative and absolute quantification (iTRAQ) or tandem mass tagging TMT multiplex labeling, one of the stable isotope labeling-based proteomic methods using LC-MS/MS, and bioinformatics analysis, are powerful methodologies for identifying novel networks and/or pathways important in biological processes/events and diseases [23,24,25,26]. These proteomic approaches are also currently becoming important tools for identifying biomarkers and host proteins involved in the pathogenicity and immune responses following viral infections [27,28,29,30]. The significance of using these proteomic approaches to evaluate the safety of virus vaccines is emerging as well [31].

The nucleus is an important cellular organelle containing most of the genetic material and a large variety of proteins such as histones and transcriptional factors to regulate gene expression. Our previous studies have demonstrated that attenuated mutants of hMPV with mutations in the motifs of 29-DEMI-32 or 39-KEALSDGI-46 enhance the activation of transcriptional factors including NF-ĸB and IRF-3. In this study, we aimed to compare the nuclear proteomic profile of cells infected with hMPV or its M2-2 mutants. We hypothesize that such a comparison can provide a way to evaluate the overall impact of M2-2 motifs on cellular responses and thereby enhance our knowledge of the efficacy and safety of mutants. We also hoped that the study would identify novel affected nuclear targets of hMPV, shed new insight on the key biological events following hMPV infection, and identify pathways for potential anti-hMPV strategies.

## 2. Materials and Methods

### 2.1. Cell Culture and Viral Preparation

The culturing of LLC-MK2 and A549 cells, hMPV stock preparation, and virus titration were done as previously described [13,17,32]. Confluent cells were infected with hMPV in serum-free media with 1.0 μg trypsin/mL at a multiplicity of infection (MOI) of 2. Mock-infected cells, defined as control or uninfected cells throughout the manuscript, were treated with the same concentration of sucrose and the same viral infection media.

### 2.2. Nuclear Fraction Preparation

Nuclear extracts of uninfected and infected cells were prepared using hypotonic/nonionic detergent lysis according to Schaffner’s protocol [33]. To prevent contamination with cytoplasmic proteins, isolated nuclei were purified by centrifugation through 1.7 M sucrose buffer A for 30 min at 12,000 rpm before nuclear protein extraction, as previously described [12].

### 2.3. LC-MS/MS Nuclear Protein Analysis and Data Processing

**Figure 2:**
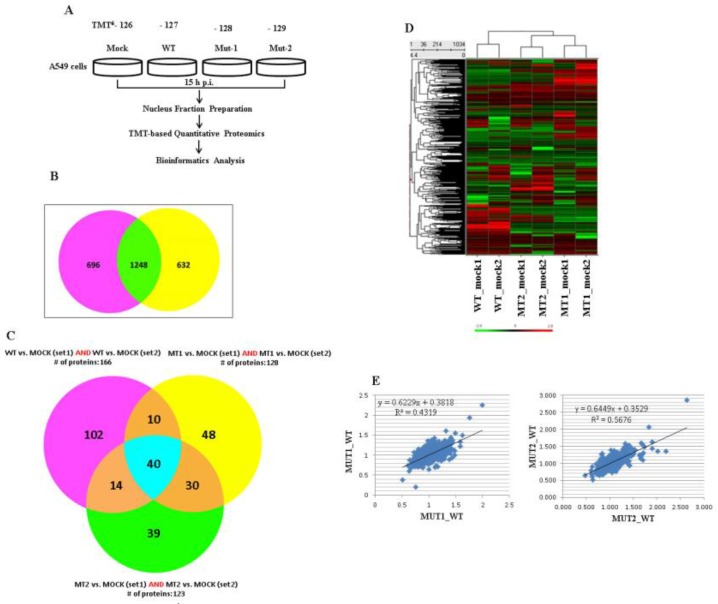
Nuclear protein expression changes with hMPV infection. (**A**) The overall workflow. A549 cells were mock infected or infected with viruses as indicated at a multiplicity of infection (MOI) of 2 for 15 h. Nuclear fractions were prepared and followed sequentially by protein quantification, reduction, precipitation, and digestion. The tandem mass tagging (TMT) isobaric mass tagging kit was then used to label the peptides followed by the fraction using a PicoFrit column. The treated samples were then subjected to LC-MS/MS analysis. Two technical sets of the samples were done at different times; (**B**) Number of detected nuclear proteins. There were 1944 and 1880 detected proteins in each experimental set. Among them, 1248 genes were identified to be common between the two sets; (**C**) Common proteins with expression fold change ≥1.2 with hMPV infection, WT, Mut-1 (MT1), or Mut-2 (MT2). There were 166 common proteins that changed with WT hMPV infections from two technical settings. In addition, 128 and 123 proteins were changed with Mut-1 and Mut-2 infections, respectively. Forty proteins were further identified to change with all three infections; (**D**) Overall nuclear protein changes in response to hMPV infection. hMPV-induced changes of nuclear proteins were obtained by comparing their abundance in infected cells to that of corresponding mock-infected samples. Heat map with hierarchical clustering was then performed for the proteins with fold changes above absolute 1 in three out of six infectious samples using complete linkage clustering method with z-score scaling; (**E**) Multivariate statistical analysis using the “R” program. The proteins, filtered as significant in response to hMPV infection at *p*-value ≤0.05 and with a fold change of 1.2 and above, compared to baseline (uninfected), were selected. The upregulated and downregulated changes by motifs, compared to WT infection, were plotted.

After the nuclear purification, the nuclear protein concentration was determined by bicinchoninic acid (BCA)-based protein assay (Thermo Scientific Pierce, Rockford, IL, USA). Approximately 100 µg of each protein sample was resuspended in 25 mM triethylammonium bicarbonate buffer, pH 7.8. The protein was reduced by adding 10 mM DTT and incubating at 50 °C for 30 min, followed by carbamidomethylation achieved by adding 25 mM iodoacetamide and incubating the mixture in the dark for 1 h. The proteins were precipitated by adding four volumes of precooled acetone and incubating at −20 °C overnight. The protein was pelleted at 14,000 rpm for 10 min at 4 °C. The protein pellet was then dissolved in 25 mM triethylammonium bicarbonate buffer followed by digestion with trypsin (Sigma, St. Louis, MO, USA) at a protein/enzyme ratio of 25:1. A tandem mass tag (TMT) labeling kit (TMTsixplex (TMT^6^)) (product number: 90,061, Thermo Fisher Scientific, Waltham, MA, USA) was used to label the peptides according to the manufacturer’s recommended conditions. As shown in Figure 2A, the mock sample was labeled with TMT^6^-126, wild type (WT) with TMT^6^-127, Mutant1 (MT1) with TMT^6^-128, and Mutant2 (MT2) with TMT^6^-129. After labeling and quenching, the four samples were mixed. The peptide mixtures were separated by reversed-phase liquid chromatography using an Easy-UPLC equipped with an autosampler (Thermo Fisher Scientific). A PicoFrit 150-mm × 75-mM, 5-µm particle size analytical column (New Objective, Ringoes, NJ, USA) was used for the reversed-phase liquid chromatography with a 275 min gradient (solvent A, 0.1% formic acid in water; solvent B, 0.1% formic acid in acetonitrile). A total of 5 to 30% of solvent B was used for separating the peptides. The QExactive mass analyzer was set to acquire data at a resolution of 35,000 in full scan mode and 17,500 in MS/MS mode. The top 15 most intense ions in each MS survey scan were automatically selected for MS/MS. Proteins were identified with the Proteome Discoverer (PD) 1.4 platform (Thermo Fisher Scientific) using the Sequest HT search engine that employs the UniProt mouse.fasta database with 51,532 peptide sequence entries (released July 2014). Sequest search parameters were used as follows: carbamidomethylation of cysteine and TMT^6^ modification of peptide N-terminus and lysine were set as fixed modifications and oxidation of methionine and deamination of asparagine and glutamine as variable modifications; trypsin was selected as the protease and up to two missed cleavages were used. Mass tolerance for the precursor ions was 10 ppm and for the MS/MS 0.05 Da. Peptides were filtered for a maximum false discovery rate of 1%. Protein quantification was also through PD 1.4 using the reporter ion ratios of TMT: TMT^6^-127/TMT^6^-126 (WT_Mock), TMT^6^-128/TMT^6^-126 (MT1_Mock), and TMT^6^-129/TMT^6^-126 (MT2_Mock) for each set. At least one unique peptide with a posterior error probability of <0.05 was accepted for quantification and proteins were grouped.

### 2.4. Ingenuity Pathway Analysis

To study the biological functions and pathways regulated by hMPV M2-2 PDZ binding motifs, protein expression that was significantly regulated (*p* < 0.05) was analyzed with the ingenuity pathway analysis (IPA). Biological functions were used to identify significantly regulated protein sets. Because regulation of any given protein could be a statistical anomaly (i.e., false positive), bioinformatics analyses were developed under the assumption that regulation, which is important for function, will occur in a coordinated fashion at multiple targets within a given pathway. Thus, the IPA analysis assesses over-representation of multiple targets within known pathways.

### 2.5. Western Blot Analysis

Total nuclear fractions from uninfected and infected A549 cells were quantified by the Bradford protein assay (Bio-Rad, Hercules, CA, USA), subjected to SDS-PAGE, and transferred to polyvinylidene difluoride membranes. Membranes were blocked with 5% milk in TBS-Tween and incubated with primary antibodies according to the manufacturer’s instructions. The primary antibodies for NALP4, ZYX, PMSA4, Histone H3, Histone H3K4Me2, and Histone H3K27Me3 were obtained from NeoBiolab (Cambridge, MA, USA). The antibody against RSV was from Bio-Rad (Hercules, CA, USA). Appropriate peroxidase-conjugated secondary antibodies (Santa Cruz Biotechnology, Dallas, TX, USA) were used after primary antibody incubation. Proteins were detected by autoradiography using ECL or ECL plus (Amersham Pharmacia Biotech, Little Chalfont, UK) according to the manufacturer’s protocol. Equal loading of proteins was evaluated by stripping and reprobing the membranes with Lamin B antibody.

## 3. Results

### 3.1. Experimental Design and Overview of the Quantitative Proteomic Data

A549 cells are a widely used human airway epithelial cell model for many airway infectious pathogens including hMPV [34,35,36]. We have previously shown that hMPV activates one of the most important innate immune pathways, namely the RIG-I-MAVS-TRAF pathway, in infected airway epithelial cells [12,16,36,37,38,39,40], and M2-2 uses its two putative PDZ motifs to counteract host antiviral responses [13]. As mentioned in the Introduction, recombinant hMPV with mutations in the 29-DEMI-32 (Mut-1) or 39-KEALSDGI-46 (Mut-2) motifs are promising vaccine candidates [10]. However, comprehensive studies on the impact of motifs on host responses are still needed for translational application of mutants. Herein, we first explored novel nuclear event(s) resulting from enhanced MAVS-mediated antiviral signaling by M2-2 motifs, and further evaluated the overall effects of mutants on host responses. We profiled and compared the expression levels of nuclear proteins from a group of cells infected with WT rhMPV, Mut-1, or Mut-2. In brief, LC-MS/MS was done for two technical sets of the nuclear fraction samples at 15 hours post infection, a time point where anti-hMPV signaling is significantly initiated and the number of dead infected cells is minimal [36]. Similar to WT, mutants could infect cells very well (Supplementary Materials Figure S1). Because we conducted a subcellular proteomic study, it was important to find organelle protein markers to confirm organelle purity. As shown in Supplementary Materials Figure S2, the nuclear fractions from sucrose purification reached high purity as they lacked a cytoplasmic protein nitric oxide synthase 1 (NOS1), but were enriched with nuclear protein Lamin B. Housekeeping nuclear protein markers, which were among the group of unchanged proteins, can also serve as reference controls. Indeed, many nuclear protein markers were found to be unchanged, including the nuclear protein prelamin (average ratios were 1.136, 1.009, and 1.035 in cells infected with WT, Mut-1, and Mut-2, respectively) and heterogeneous nuclear ribonucleoprotein H (average ratios were 0.9305, 0.995, and 1.000 in cells infected with WT, Mut-1, and Mut-2, respectively). This also proves that the nucleus purification method itself did not introduce bias. Our subcellular and quantitative proteomic analysis workflow is outlined in Figure 2A. Fold changes were calculated by normalizing protein expression in virus-infected cells with corresponding protein expression in mock-infected cells. Venn analysis revealed 1248 common proteins in all infected samples of both technical sets (Figure 2B). Based on the Gaussian distribution of the quantitative ratio (mean and standard deviation of 1.96 based at log_2_ value), we defined the significantly changed ratio threshold as a fold-change of 1.2. We also applied the method used by Barderas et al. and Tan et al., and confirmed the fold induction change ≥1.2 as significant [41,42]. There were 40 common proteins with fold-changes greater than absolute 1.2 (upregulated or downregulated) by viral infections in two sets (Figure 2C). Differentially expressed proteins were clustered on the expression profile using the hierarchical clustering method to help visualize patterns of protein expression within and across clusters. There were 1034 proteins with a fold change above absolute 1 in three of six infected samples selected for the clustering. The complete linkage clustering algorithm was performed on z-score scaling expression values. Color corresponds to the expression level of the transcript with low, intermediate, and high expression represented by green, black, and red, respectively. As shown in Figure 2D, we found that the expression patterns of two technical sets were quite reproducible, which was also confirmed by multivariate statistical analysis using the “R” program, a method commonly applied for metabolic profiling and cancer marker discovery by others and us (Figure 2E) [43,44]. Overall, all these results suggest that M2-2 motifs caused significant changes in nuclear protein abundance.

### 3.2. Experimental Data Validation

**Figure 3:**
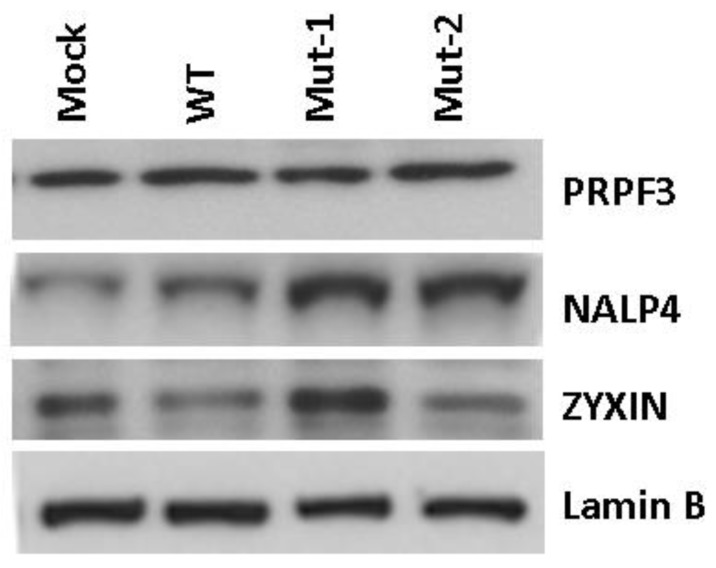
Experimental confirmation of the nuclear protein changes with hMPV infection. Total nuclear fractions prepared from A549 cells uninfected or infected with rhMPV (MOI of 2) were resolved on 10% SDS-PAGE and Western blot was performed using antibodies against PRPF3, NALP4, and zyxin. Membranes were stripped and reprobed for Lamin B as an internal control for protein integrity and loading.

To validate the proteomics data, we used Western blot to analyze three proteins with different expression patterns in response to WT and mutant virus infections (Figure 3). The Western blot results determined PRPF3 (pre-mRNA processing factor 3), a U4/U6 small nuclear ribonucleoprotein, was not affected by mutations, which is in accordance with our proteomics analysis. The Western blot also confirmed that nuclear NALP4 (NACHT, LRR, and PYD domains containing protein 4) was significantly enhanced by WT virus infection. The enhanced expression was further increased in Mut-1- and Mut-2-infected cells. NALP4 is a known cytosolic protein of inflammasomes [45], but our current study suggests its presence in the nucleus as well. According to the proteomics data, zyxin, a zinc-binding phosphoprotein, was significantly decreased following WT and Mut-2 infection. However, Mut-1 infection led to a marginal change. This was also confirmed by Western blot, suggesting reliable quantitative proteomics for unbiased discovery of novel cellular responses to hMPV infection.

### 3.3. Nuclear Proteins Regulated by M2-2 Motifs

**Figure 4:**
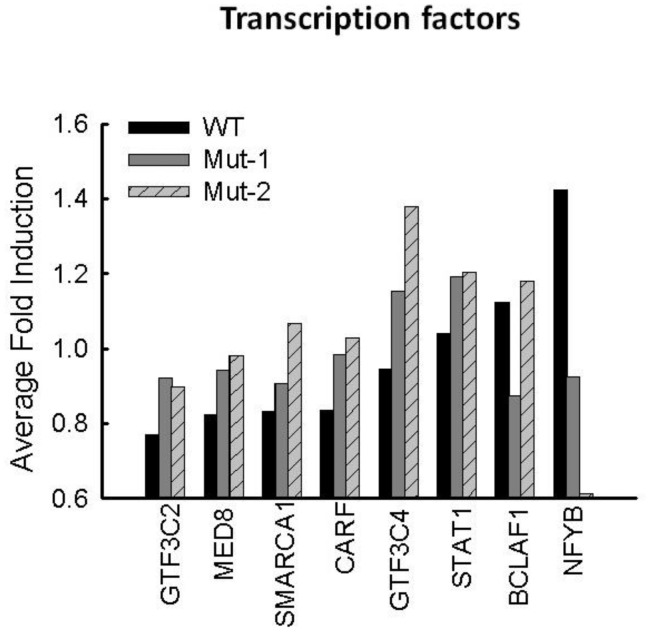
Nuclear transcription factors affected by M2-2 motifs. Detected transcription factors were analyzed as described in the Supplementary Table S1. Motif-affected transcriptional factors are summarized and presented.

We have shown previously that hMPV-induced nuclear translocation of transcription factors NF-ĸB and IRF-3 is significantly inhibited by the motifs 29-DEMI-32 and 39-KEALSDGI-46 via their disruption of the MAVS–TRAF interaction. To further investigate whether other transcription factors are affected by the motifs, we compared their abundance in WT- and mutant-infected cells. As shown in Supplementary Table S1, there were 25 transcriptional factors detected in all samples. Among those, the abundance of 17 transcriptional factors was not affected or marginally affected by infections. Among eight affected nuclear transcription factors, GTF3C2 (general transcription factor IIIC subunit 2), MED8 (mediator complex subunit 8), SMARCA1 (SWI/SNF related, matrix associated, actin dependent regulator of chromatin, subfamily A, member 1), and CARF (calcium-responsive transcription factor) were significantly decreased by WT infection. However, the decrease was rescued by both mutant infections, suggesting that hMPV requires both PDZ motifs to downregulate the activities of these transcriptional factors during the infection. Unlike GTF3C2, we found that the expression of GFT3C4 (general transcription factor IIIC subunit 4) was not affected by WT infection. However, Mut-1 infection (mutations in the 29-DEMI-32 motif) and Mut-2 infection (mutations in the 39-KEALSDGI-46 motif) resulted in increased expression of GFT3C4, with Mut-2 infection having more impact. We also discovered that WT infection led to more nuclear localized NFYB (nuclear transcription factor Y subunit beta). Conversely, the nuclear presence was significantly reduced by Mut-2 infection. In addition, motifs 29-DEMI-32 and/or 39-KEALSDGI-46 also played a role in regulating the nuclear presence of BCLAF1 (BCL2-associated transcription factor 1). These transcription factors are more or less associated with RNA polymerase transcription and functions (GTF3C2, MED8, and CARF), chromatin remodeling (SMARCA1), and sequence-specific DNA binding (NYFB and SMARCA1), suggesting the importance of motifs 29-DEMI-32 and 39-KEALSDGI-46 in mediating these events [46,47,48,49,50]. The fold changes of impacted proteins are summarized in Figure 4.

**Figure 5:**
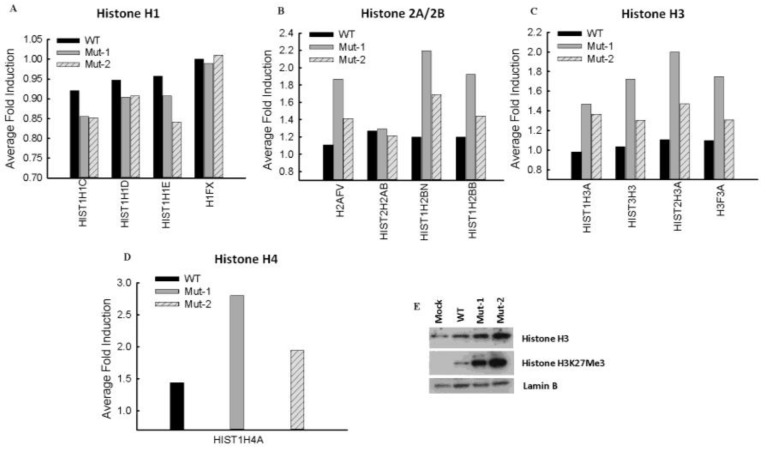
Changes in nuclear histone proteins in response to hMPV infection. Nuclear histone proteins were analyzed as described in the Supplementary Table S1. Motif-affected histone 1 (**A**), histone 2 (**B**), histone 3 (**C**), and histone 4 (**D**) are summarized and presented. The changes and methylation levels of histone 3 by hMPV were investigated by Western blot using antibodies against the indicated proteins with/without protein modification (**E**).

Histone proteins are key components of chromatin, acting as spools around which DNA winds, and play a significant role in gene regulation. Additionally, their function in viral infections is being increasingly acknowledged [51,52,53,54]. Currently, there are five major families of histones: H1/H5, H2A, H2B, H3, and H4. Histones H2A, H2B, H3, and H4 are known as the core histones, while histones H1.0-H1.5 are known as the linker histones (https://www.ncbi.nlm.nih.gov/projects/HistoneDB2.0/). As shown in Figure 5A, the overall detected H1 proteins were not affected by infection with wild type or either mutant as their changes after normalizing the expression to mock-infected samples were not significant (0.83 < fold induction < 1.2). There were four detected H2 proteins in all samples of both technical replicates (Figure 5B), among which two H2A proteins (H2AFV and H2AB) and two H2B proteins (H2BN and H2BB), that were impacted by WT, Mut-1, and/or Mut-2 infections. The expression of H2AFV was not influenced by WT infection but was enhanced by Mut-1 and Mut-2 infections. The nuclear abundance of three other H2 proteins was increased by WT infection. For H2AB, the two motifs did not play a role in the protein increase. However, the motifs appear to have suppressed the enhancement of H2BN and H2BB with motif 29-DEMI-32 having a more suppressive function than motif 39-KEALSDGI-46. We also found that viruses with mutations in either motif resulted in more H3 proteins in the nucleus compared to WT infection (Figure 5C). In this study, we detected only one common detectable H4 protein, H4A, in all samples. As shown in Figure 5D, the nuclear abundance of H4A was significantly increased by WT infection, and the mutations in M2-2 motifs resulted in increased H4A expression. Many viruses, including human cytomegalovirus, vaccinia virus, and herpes simplex virus regulate H3 protein expression or modify H3 methylation to change viral latency or replication [53,55,56]. Therefore, in this study, we selected H3 as a target to validate the effect of motifs on the summarized histone protein expression. As shown in Figure 5E, both motifs are important for H3 expression. Given the fact that H3 methylation plays a critical role in host responses to viral infections including RSV [53,57], we also investigated the effects of the motifs on the methylation status of the H3 residues K27 and K4. Methylation was detectable only on H3K27, not H3K4, and, therefore, regulated by the M2-2 motifs. The overall summary of histone protein changes with parameters listed in detail in Supplementary Materials Table S2.

### 3.4. Nuclear Viral Protein

**Figure 6:**
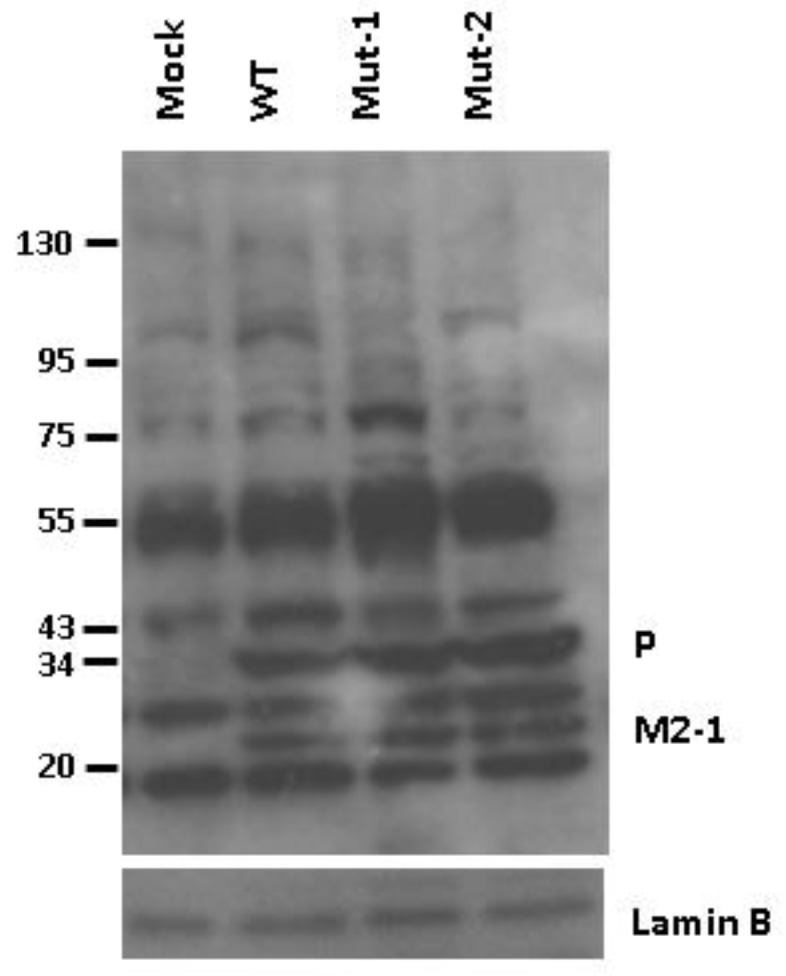
Nuclear presence of hMPV proteins. Cell infection was done as described in Figure 1. The nuclear presence of hMPV proteins was determined by Western blot using an antibody against hMPV. The membrane was stripped and reprobed using an antibody against Lamin B as an internal control for protein integrity and loading.

As a negative-sense RNA virus, the viral genome replication and gene transcription of hMPV are believed to occur in the cytoplasmic compartment [58]. Surprisingly, our proteomic studies revealed the presence of two viral proteins, P and M2-1, in the nucleus, which was confirmed by Western blot (Figure 6). However, the presence of P and M2-1 seemed unaffected by the M2-2 motifs.

### 3.5. Motif-Regulated Pathways Identified by IPA

**Figure 7:**
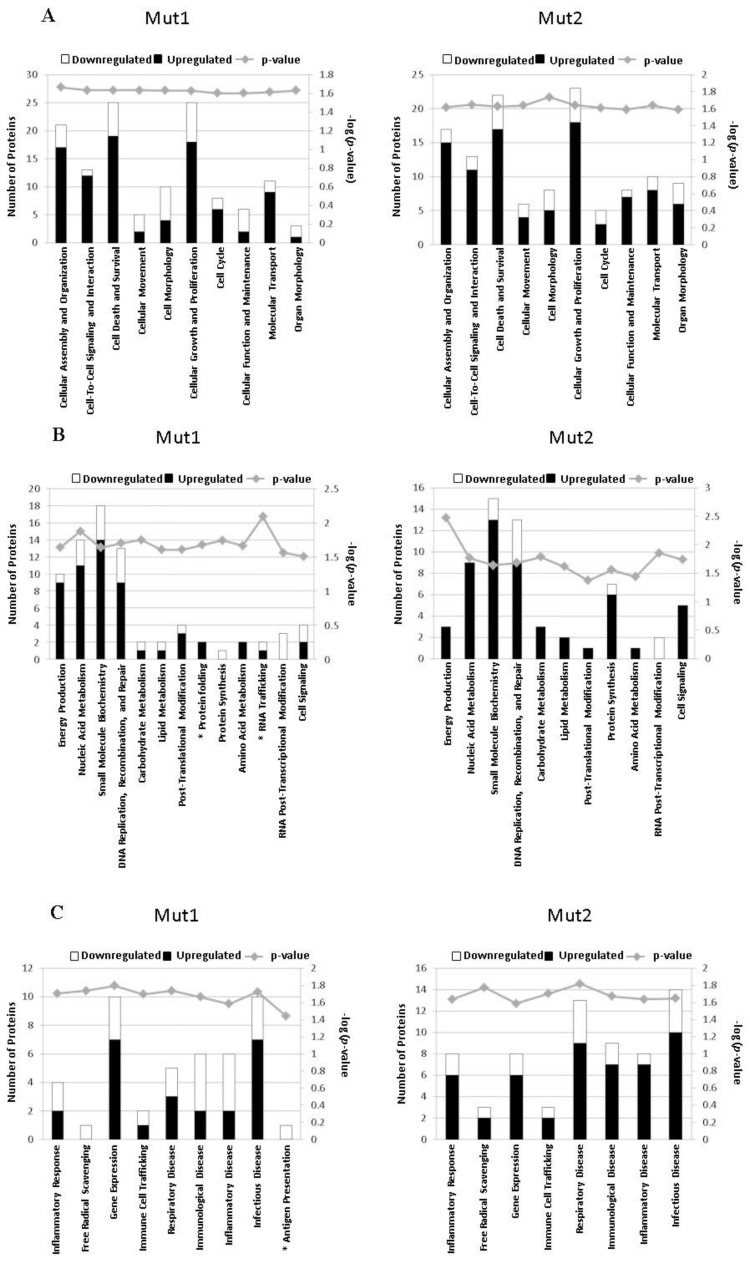
Ingenuity pathway analysis (IPA) of the dataset consisting of the hMPV-regulated proteins determined by mass spectrometry. The left Y-axis is the number of proteins associated with significant changes by M2-2 motifs. The proteins were identified in this study over whole known proteins in the literature in the pathways and its value is shown by the height of each bar. The right Y-axis is the negative log (*p*-value) representative of the significance of the pathway and its value is shown by the gray square and linked by the gray line within pathways. Representative pathways belonging to cell functions (**A**), metabolism (**B**), and infection and immune response (**C**) are selected and shown.

We also used the current knowledge-based database in IPA to identify motif-regulated proteins and their associated responses. We chose the biological process classification for the data process as there are many infection-related sub-classifications. As shown in Figure 7A, both motifs influenced the cellular functions through proteins that are involved in cellular growth and proliferation, cell death and survival, and cellular assembly and organization, etc. All of these cellular functions have been reported to be more or less associated with viral replication [59,60,61]. In addition, there were significant quantities of proteins involved in nucleic acid metabolism, DNA repair, and immune and inflammatory responses (Figure 7B,C). We also found that Mut-1 infection affected some unique biological functions, such as antigen presentation, RNA trafficking, and protein folding (marked with an asterisk in the left panels of Figure 7B,C). Some common pathways were regulated by motifs of both mutants in a similar way. For example, both motifs suppressed the nuclear abundance of CCT proteins, SUPT16H, and histone 3H3 to affect cellular assembly and organization (Supplementary Table S3). Sometimes, each motif differentially used targets to influence the same pathways. One example is HMGB1 (high mobility group box 1), a DNA binding protein critical for the regulation of type I interferon and inflammatory responses in viral infection [62,63]. Our analysis indicates that HGBM1 is involved in 28 biological functions (Supplementary Materials Table S3). In terms of the expression of HGBM1, it was significantly reduced by Mut-1 infection, while WT and Mut-2 infections did not influence the nuclear abundance of HMGB1. The molecular mechanisms underlying the regulation of HGBM1 by the motif 29-DEMI-32 and the associated biological consequence are unknown but will be investigated in the near future. Another motif-specific regulatory target was PRDX1 (peroxiredoxin 1), a molecule predicted by IPA to be involved in 17 biological functions including free radical scavenging, inflammatory diseases, and infectious diseases (Supplementary Table S3). We found that WT infection did not affect the nuclear presence of PRDX1. However, its abundance was significantly decreased by Mut-1 infection but, interestingly, increased by Mut-2 infection (Supplementary Materials Table S3). Although PRDX1 has been reported to be involved in viral gene transcription and replication [64], we do not think that PRDX1 plays a role in mediating hMPV replication and gene transcription given the fact that the mutations in either motif lead to attenuated replication and gene transcription [13]. The differential regulation of PRDX1 by motif 29-DEMI-32 and motif 39-KEALSDGI-46 may lead to different inflammatory and stress responses to WT, Mut-1, and Mut-2 infections, as PRDX1 is an important regulator in these responses [65]. In the future, we will address whether PRDX1 is important in mediating hMPV-induced inflammatory and oxidative responses and how PRDX1 is regulated by the motifs. This will also provide a base to evaluate the safety of these vaccine candidates. We also found that NCOR1 (nuclear receptor corepressor 1) expression was sensitive only to Mut-2 infection (Supplementary Materials Table S3). It would be of interest to study the role of NCOR1 in hMPV infection and its regulation by the PDZ motif 39-KEALSDGI-46.

## 4. Discussion

Over the past decade, proteomic approaches have become useful tools for the discovery and understanding of host–pathogen interactions that represent anti- and pro-pathogenic or immunogenic responses. Here, we used the TMT labeling technology to quantify changes in nuclear proteins by two hMPV motifs, namely 29-DEMI-32 and 39-KEALSDGI-46. We have previously found that mutants with either motif mutated have attenuated replication suggesting that both motifs promote hMPV replication [13]. We also found that the motifs uncouple the interaction between MAVS and its downstream effectors (TRAFs), leading to disruption of RIG-MAVS antiviral signaling and subsequently inhibiting the nuclear translocation of two transcription factors belonging to the NF-ĸB and IRF families. However, the impact of motifs on other nuclear transcription factors has not been explored.

Herein, we initiated the investigation to define alterations in nuclear protein abundance and found that there were significant changes in the nuclear transcription factors. As discussed, some affected transcription factors are involved in RNA polymerase transcription and functions (GTF3C2, MED8, and CARF). RNA polymerase II has been shown to be highly involved in antiviral responses. For example, herpes simplex virus regulates the nuclear abundance of RNA polymerase II to repress the host gene transcription [66]. Another example is that Bunyamwera orthobunyavirus NS proteins interact with MED8 to block RNA polymerase II activity and thereby counteract host antiviral responses [67]. In this study, several transcriptional factors responsible for the transcription of RNA polymerase II were decreased in response to WT hMPV, but the decrease was rescued by Mut-1 or Mut-2 infection. In the future, we will study whether hMPV uses these two motifs to suppress overall host gene transcription via inhibiting RNA polymerase abundance or activity through these transcription factors.

Chromatin remodeling is a key immune or pathogenic mechanism used by the host to respond to viral infections [68,69,70]. As discussed, not only were some transcription factors involved in chromatin remodeling (SMARCA1), but also many histones themselves, especially the core histones, were found to be significantly impacted by the M2-2 motifs. Some viruses use their viral proteins to directly regulate the functions of histones. For example, the capsid protein of the Dengue virus binds to the four cellular core histones to disrupt normal host cell genetic machinery in favor of viral replication and the virus lifecycle [71]. Another example is the latency-associated nuclear antigen of the Kaposi’s sarcoma-associated herpesvirus, which upregulates H2A to control its infectivity [72]. However, chromatin regulation by M2-2 did not likely result from direct M2-2–histone interaction, as we did not detect M2-2 in the nucleus. It is possible that M2-2 regulates histone expression via the innate signaling. Innate signaling, such as IFN-γ, has been reported to regulate histone abundance in some viral infections [73]. It is increasingly recognized that the histone epigenetic mechanisms are highly associated with host responses to viral infections [74,75]. Our results have also demonstrated that M2-2 can modify histone methylation (Figure 5). Although the roles of histones in hMPV infection have not been studied, given the importance of histones in the regulation of innate cytokine induction and pathogenesis in response to respiratory syncytial virus, a close family member of hMPV, it is likely that they are critical in hMPV infection [57,76]. In the future, we will confirm their role by using inhibitors or siRNAs to specifically control the activities or abundance of histones or their modification enzyme(s). We will also study the mechanism(s) underlying the M2-2-regulated histone expression and modification.

Other than transcription factors and histones, we also found that some proteins, such as zyxin and NALP4, whose functions are unclear in virus infections, were affected by the motif 29-DEMI-32 and/or 39-KEALSDGI-46. Zyxin has been reported to be essential for tight cell-to-cell junctions and for modulating the transmigration of *Haemophilus influenzae* to the central nervous system [77]. Since hMPV infection is restricted in the airway tract, it is unlikely for zyxin to carry out such a function in the context of hMPV infection. Recently, zyxin was reported to stabilize RIG-I–MAVS interaction and promote type I IFN response [78]. Since RIG-I-MAVS signaling is critical in hMPV-induced innate responses, it is worthwhile investigating in the near future whether zyxin serves as a scaffold for the interactions between RIG-I and MAVS and how M2-2 motifs regulate zyxin shuttling between the nuclear and cytosolic compartments. As shown in Figure 3, the nuclear induction of NALP4 was significantly impacted by PDZ motifs. NALP4 is thought to be a cytosolic protein of inflammasomes, but has not been studied as deeply as its family member NALP3 [45]. In the future, we will investigate its role in hMPV-induced inflammatory responses.

As mentioned, wild-type recombinant hMPV has been approved as a suitable parent virus for the development of live attenuated hMPV vaccine candidates in experimental human infection trials. Supporting live attenuated vaccine development is a promising research direction [8]. More related to our M2-2-based hMPV vaccine development, the M2-2-deleted RSV, a close family member of hMPV, is currently being tested clinically [79]. Our M2-2-based mutants are promising because of their attenuation, intact CTL epitope, balanced expression of F and G proteins, and ability to induce stronger immunity. This study has discovered several novel molecules and pathways that are affected by hMPV M2-2 PDZ motif(s). Whether the molecules/pathways are important for host immunity and pathogenesis needs to be further characterized. However, they will give new insight into the attenuation mechanisms of M2-2 mutants and will subsequently provide a base on which to evaluate the safety of vaccine candidates, as well as provide strategies for enhancing vaccine efficacies by modifying the expression or activities of affected host molecules.

## 5. Conclusions

In summary, this study provided deep insight into the overall and novel impact of M2-2 PDZ binding motifs on cellular responses via an unbiased quantitative proteomic analysis and comparison. Several key pathways and nuclear proteins controlled by M2-2 PDZ domain-binding motif(s) were identified. The results are critical and valuable for evaluating M2-2 mutants as potent vaccine candidates.

## Supplementary Materials

The following are available online at www.mdpi.com/2076-393X/5/4/45/s1. Supplementary Figure S1: The impact of M2-2 motifs on the expression of viral proteins. Supplementary Figure S2: Purity of isolated nuclear compartments. Supplementary Table S1: Nuclear expression of transcription factors. Supplementary Table S2: Nuclear expression of histone proteins. Supplementary Table S3: Motif-regulated biological functions identified by IPA.
