# Supplementary material for: Detection of Nuclear Protein Profile Changes by Human Metapneumovirus M2-2 Protein Using Quantitative Differential Proteomics
Source: Vaccines (Basel). 2017 Dec 3;5(4):45. doi: 10.3390/vaccines5040045 (PMC5748611; doi:10.3390/vaccines5040045)
Supplement: Supplementary file 1 [file vaccines-05-00045-s001.pdf]

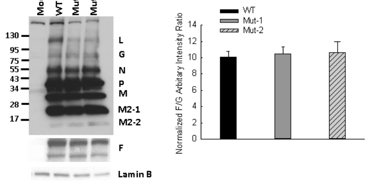
Mock
WT
Mut-1
Mut-2
130
95
75
55
43
34
28
17
L
G
N
P
M
M2-1
M2-2
F
Lamin B
WT
Mut-1
Mut-2
Normalized F/G Arbitary Intensity Ratio
14
12
10
8
6
4
2
0

Figure S1.

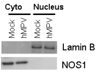
Cyto
Nucleus
Mock
hMPV
Mock
hMPV
Lamin B
NOS1

WT/mock	Mut-1/mock	Mut-2/mock	
**0.771**	0.921	0.898	**GTF3C2**
**0.824**	0.944	0.983	**MED8**
**0.834**	0.907	1.067	**SMARCA1**
**0.837**	0.984	1.028	**CARF**
0.850	1.048	0.963	NFYC
0.858	0.931	1.005	TRRAP
0.906	0.976	0.944	SUPT5H
0.908	0.910	0.938	GTF2I
0.930	0.907	0.878	TRIM28
0.946	**1.153**	**1.379**	**GTF3C4**
0.961	0.997	1.019	SMARCA2
0.967	0.965	0.903	GTF2H4
0.987	0.928	0.898	YY1
0.996	0.930	0.952	SLTM
0.998	0.948	0.943	TCERG1
1.007	0.946	0.929	BTF3
1.019	0.875	0.904	UBTF
1.026	1.013	0.929	GTF3C1
1.029	0.992	0.980	GTF3C3
1.031	1.012	1.036	MED11
1.040	**1.192**	**1.205**	**STAT1**
1.111	1.100	0.939	CTCF
1.124	0.874	**1.181**	**BCLAF1**
1.129	1.058	0.952	TAF8
**1.424**	0.925	**0.613**	**NFYB**

		Set 1 Score	Coverage	WT	Mut-1	Mut-2	Set 2 Score	Coverage	WT	Mut-1	Mut-2
P16403	Histone H1.2 HIST1H1C	3016.70	100.00	0.865	0.827	0.839	2431.89	99.06	0.977	0.884	0.865
P16402	Histone H1.3 HIST1H1D	2877.30	92.31	0.943	0.893	0.872	2287.59	99.10	0.952	0.916	0.943
P10412	Histone H1.4 HIST1H1E	3175.00	100.00	0.909	0.900	0.847	2588.09	99.09	1.006	0.915	0.834
Q92522	Histone H1x H1FX	566.19	97.65	0.990	0.990	0.998	464.44	100.00	1.010	0.988	1.024
Q71UI9	Histone H2A.V H2AFV	301.46	97.66	0.980	1.867	1.407	248.72	82.03	1.238	1.870	1.422
Q8IUE6	Histone H2A type 2-B HIST2H2AB	704.55	93.08	1.298	1.265	1.186	326.12	94.62	1.242	1.323	1.243
U3KQK0	Histone H2B HIST1H2BN	1267.13	87.95	1.174	2.265	1.699	936.64	87.95	1.224	2.126	1.679
P33778	Histone H2B type 1-B HIST1H2BB	1190.07	100.00	1.184	2.001	1.499	878.92	97.62	1.210	1.840	1.388
P68431	Histone H3.1 HIST1H3A	346.26	88.24	0.911	1.517	1.449	250.65	90.44	1.047	1.419	1.282
Q16695	Histone H3.1t HIST3H3	325.08	88.24	0.986	1.611	1.222	236.19	90.44	1.084	1.835	1.381
Q71DI3	Histone H3.2 HIST2H3A	473.92	88.24	1.136	1.952	1.484	353.05	90.44	1.075	2.042	1.456
P84243	Histone H3.3 H3F3A	433.65	88.24	1.043	1.738	1.273	332.29	97.06	1.146	1.756	1.340
P62805	Histone H4 HIST1H4A	737.96	100.00	1.400	2.829	1.901	550.10	95.15	1.478	2.774	1.985

© 2000-2015 QIAGEN. All rights reserved.

Biological Functions	Analysis Name	*p*-value	Molecules	# of proteins
Cellular Assembly and Organization	Mut1 vs. Mock1 & Mut1 vs. Mock2	4.15E-13-4.33E-02	CCT2,CCT3, CCT4,CCT5,CCT6A,CCT7,CCT8,GAPDH,GNB2L1, HIST3H3,MYOF,NUP155,SUPT16H,TCP1,SET, BASP1,*FAM109B*,HMGN1,HMGB1,	20
	Mut2 vs. Mock1 & Mut2 vs. Mock2	5.13E-06-4.85E-02	ANXA1,CCT3,CCT5,CCT6A,CCT7,CCT8,EEF1A1,GAPDH,HIST3H3,KRT9,MYOF,PLXNB1,SLC25A5,SSRP1,SUPT16H, HIST1H1C,NPM1	17
Cell-To-Cell Signaling and Interaction	Mut1 vs. Mock1 & Mut1 vs. Mock2	1.87E-12-4.65E-02	CCT2,CCT3,CCT4,CCT5,CCT6A,CCT7,CCT8,CNN2,GAPDH,GNB2L1,MYOF,TCP1, HMGB1	13
	Mut2 vs. Mock1 & Mut2 vs. Mock2	1.04E-05-4.51E-02	ANXA1,CCT3,CCT5,CCT6A,CCT7,GAPDH,KRT2,MYOF,OASL,PLXNB1,NPM1,PAWR	12
Cell Death and Survival	Mut1 vs. Mock1 & Mut1 vs. Mock2	1.78E-09-4.65E-02	ATP5A1,CCT2,CCT3,CCT4,CCT5,CCT6A,CCT7,CCT8,CNN2,EIF6,GAPDH,GNB2L1,MYOF,ORC2,SET,SND1,TCP1,USP10, BASP1,CHTOP,HDGF,HMGB1,PQBP1,PRDX1	24
	Mut2 vs. Mock1 & Mut2 vs. Mock2	7.41E-05-4.74E-02	ANXA1,BCLAF1,CCT3,CCT5,CCT6A,CCT7,EEF1A1,GAPDH,G6PD,KRT10,MYOF,PLXNB1,PRDX1,SET,SLC25A5,SSRP1,TPT1, HDGF,NPM1,PAWR	20
Cellular Movement	Mut1 vs. Mock1 & Mut1 vs. Mock2	7.21E-05-4.65E-02	CNN2,GNB2L1,HDGF,HMGB1,HMGB3	5
	Mut2 vs. Mock1 & Mut2 vs. Mock2	4.45E-04-4.55E-02	ANXA1,PLXNB1,PRDX1,SSRP1,HDGF,NPM1	6
Cell Morphology	Mut1 vs. Mock1 & Mut1 vs. Mock2	2.36E-04-4.65E-02	CNN2,GNB2L1,SET,BASP1,CHTOP,DPY30,ERH,HMGB1,PRDX1	9
	Mut2 vs. Mock1 & Mut2 vs. Mock2	3.1E-03-3.36E-02	ANXA1,KRT2,PLXNB1,PRDX1,DPY30,NCOR1,NPM1	7
Cellular Growth and Proliferation	Mut1 vs. Mock1 & Mut1 vs. Mock2	6.16E-04-4.65E-02	ATP5A1,ATP5B,ATP5G1,CCT2,CCT3,CCT5,CCT7,CNN2,EIF6,GAPDH,GNB2L1,MYOF,NAP1L1,SET,SND1,TCP1,USP10, BASP1,DPY30,HDGF,HMGB1,HMGB3,HMGN1,PRDX1	24
	Mut2 vs. Mock1 & Mut2 vs. Mock2	3.1E-03-4.27E-02	ATP5G1,ANXA1,BCLAF1,CCT3,CCT5,CCT7,EEF1A1,GAPDH,G6PD,KRT2,KRT10,MYOF,NAP1L1,PLXNB1,PRDX1,SET,SLC25A5,TPT1, DPY30,HDGF,NCOR1,NPM1,PAWR	23
Cell Cycle	Mut1 vs. Mock1 & Mut1 vs. Mock2	3.4E-03-4.65E-02	*BANF*1,CCT4,GNB2L1,ORC2,SUPT16H,TCP1, HMGB1,HMGN1	8
	Mut2 vs. Mock1 & Mut2 vs. Mock2	4.45E-04-4.85E-02	*BANF1*,SSRP1,SUPT16H,NPM1,PAWR	5
Cellular Function and Maintenance	Mut1 vs. Mock1 & Mut1 vs. Mock2	3.4E-03-4.65E-02	MYOF, NUP155,HMGB1,HMGN1,PRDX1	5
	Mut2 vs. Mock1 & Mut2 vs. Mock2	2.93E-03-4.85E-02	ANXA1,GAPDH,KRT9,MYOF,PLXNB1,PRDX1,SSRP1,NPM1	8
Molecular Transport	Mut1 vs. Mock1 & Mut1 vs. Mock2	3.4E-03-4.54E-02	ATP5B,ATP5L,GAPDH,GNB2L1,NUP155,SET,SLC25A15,CHTOP,HMGB1	9
	Mut2 vs. Mock1 & Mut2 vs. Mock2	6.12E-04-4.55E-02	ANXA1,EEF1A1,GAPDH,G6PD,PRDX1,SET,SLC25A5,TPT1,DPY30,PAWR	10
Organ Morphology	Mut1 vs. Mock1 & Mut1 vs. Mock2	3.4E-03-4.33E-02	MYOF,HDGF,HMGB1	3
	Mut2 vs. Mock1 & Mut2 vs. Mock2	3.1E-03-4.85E-02	ANXA1,BCLAF1,G6PD,MTHFD2,MYOFHDGF,NCOR1,NPM1	8
Energy Production	Mut1 vs. Mock1 & Mut1 vs. Mock2	4.34E-08-4.65E-02	ATP5A1,ATP5B,ATP5C1,ATP5G1,ATP5L,CCT4,CCT5, HMGB1	8
	Mut2 vs. Mock1 & Mut2 vs. Mock2	3.11E-03-3.64E-03	ATP5D,ATP5G1,SLC25A5	3
Nucleic Acid Metabolism	Mut1 vs. Mock1 & Mut1 vs. Mock2	4.34E-08-2.68E-02	ATP5A1,ATP5B,ATP5C1,ATP5G1,ATP5L,CCT4,CCT5,GAPDH,SET, ERH,HMGB1,HMGN1	12
	Mut2 vs. Mock1 & Mut2 vs. Mock2	6.12E-04-3.36E-02	ATP5D,ATP5G1,EEF1A1,ERH,GAPDH,G6PD,SET,SLC25A5,TPT1	9
Small Molecule Biochemistry	Mut1 vs. Mock1 & Mut1 vs. Mock2	4.34E-08-4.65E-02	ATP5A1,ATP5B,ATP5C1,ATP5G1,ATP5L,CCT4,CCT5,EIF6,GAPDH,GNB2L1,SET, ERH,HMGB1,HMGN1,PRDX1	15
	Mut2 vs. Mock1 & Mut2 vs. Mock2	6.12E-04-4.55E-02	ATP5D,ATP5G1,ANXA1,EEF1A1,ERH,GAPDH,G6PD,MTHFD2,PRDX1,SET,SLC25A5,TPT1,NCOR1,PAWR	14
DNA Replication, Recombination, and Repair	Mut1 vs. Mock1 & Mut1 vs. Mock2	2.12E-05-4E-02	*BANF1*,CCT4,CCT5,HIST3H3*,NAP1L1*,ORC2,SET,SUPT16H, ERH,HDGF,HMGB1,HMGN1	12
	Mut2 vs. Mock1 & Mut2 vs. Mock2	2.11E-03-3.96E-02	*BANF1*,ERH,G6PD,HIST3H3,NAP1L1,PRDX1,SET,SSRP1,SUPT16H, HDGF,NPM1,PAWR	12
Carbohydrate Metabolism	Mut1 vs. Mock1 & Mut1 vs. Mock2	3.4E-03-3.24E-02	GAPDH,HMGB1	2
	Mut2 vs. Mock1 & Mut2 vs. Mock2	3.1E-03-2.99E-02	EEF1A1,GAPDH,G6PD	3
Lipid Metabolism	Mut1 vs. Mock1 & Mut1 vs. Mock2	3.4E-03-4.65E-02	HMGB1	1
	Mut2 vs. Mock1 & Mut2 vs. Mock2	3.1E-03-4.55E-02	ANXA1,EEF1A1	2
Post-Translational Modification	Mut1 vs. Mock1 & Mut1 vs. Mock2	3.4E-03-4.65E-02	CCT4,GNB2L1,TCP1,PRDX1	4
	Mut2 vs. Mock1 & Mut2 vs. Mock2	4.26E-02-4.26E-02	ANXA1	1
Protein Folding	Mut1 vs. Mock1 & Mut1 vs. Mock2	3.4E-03-3.91E-02	CCT4,TCP1	2
Protein Synthesis	Mut1 vs. Mock1 & Mut1 vs. Mock2	6.29E-03-3.02E-02	HMGB1	1
	Mut2 vs. Mock1 & Mut2 vs. Mock2	6.76E-03-4.85E-02	ANXA1,EEF1A1,GAPDH,G6PD,PRDX1,NPM1	6
Amino Acid Metabolism	Mut1 vs. Mock1 & Mut1 vs. Mock2	6.78E-03-3.67E-02	SLC25A15	1
	Mut2 vs. Mock1 & Mut2 vs. Mock2	3.66E-02-3.66E-02	MTHFD2	1
RNA Trafficking	Mut1 vs. Mock1 & Mut1 vs. Mock2	8.16E-03-8.16E-03	NUP155,CHTOP	2
RNA Post-Transcriptional Modification	Mut1 vs. Mock1 & Mut1 vs. Mock2	8.56E-03-4.65E-02	PQBP1,RBMX,SNRPF	3
	Mut2 vs. Mock1 & Mut2 vs. Mock2	9.27E-03-1.85E-02	NPM1,RPS16	2
Cell Signaling	Mut1 vs. Mock1 & Mut1 vs. Mock2	1.69E-02-4.54E-02	GNB2L1,SET,HMGB1,PRDX1	4
	Mut2 vs. Mock1 & Mut2 vs. Mock2	3.1E-03-3.36E-02	*BANF1*,EEF1A1,MICU2,OASL,SET	5
Inflammatory Disease	Mut1 vs. Mock1 & Mut1 vs. Mock2	5.61E-03-4.65E-02	GAPDH,SND1,ERH,HMGB1,LDHB,PRDX1	6
	Mut2 vs. Mock1 & Mut2 vs. Mock2	3.8E-04-4.58E-02	ATP5G1,ANXA1,EEF1A1,GAPDH,KRT10,PRDX1	6
Free Radical Scavenging	Mut1 vs. Mock1 & Mut1 vs. Mock2	3.4E-03-3.34E-02	PRDX1	1
	Mut2 vs. Mock1 & Mut2 vs. Mock2	3.1E-03-3.06E-02	G6PD,PRDX1,DPY30	3
Gene Expression	Mut1 vs. Mock1 & Mut1 vs. Mock2	3.4E-03-2.87E-02	CCT2,GAPDH,GNB2L1,NAP1L1,SET,SND1,SUPT16H, HMGB1,HMGN1,PRDX1	10
	Mut2 vs. Mock1 & Mut2 vs. Mock2	3.1E-03-4.88E-02	GAPDH,G6PD,PRDX1,SET,SSRP1,SUPT16H, NCOR1,NPM1	8
Immune Cell Trafficking	Mut1 vs. Mock1 & Mut1 vs. Mock2	3.4E-03-3.68E-02	CNN2,HMGB1	2
	Mut2 vs. Mock1 & Mut2 vs. Mock2	3.1E-03-3.66E-02	ANXA1,PRDX1,NPM1	3
Respiratory Disease	Mut1 vs. Mock1 & Mut1 vs. Mock2	3.4E-03-3.34E-02	DNAI1,GAPDH,SND1,HMGB1,PRDX1	5
	Mut2 vs. Mock1 & Mut2 vs. Mock2	1.22E-04-3.02E-02	ANXA1,DNAI1,EEF1A1,GAPDH,KRT2,KRT10,PLXNB1,PRDX1,TPT1,AHNAK2,HIST1H1E,RPS11	12
Immunological Disease	Mut1 vs. Mock1 & Mut1 vs. Mock2	5.61E-03-3.76E-02	GAPDH,SND1,ERH,HMGB1,LDHB,PRDX1	6
	Mut2 vs. Mock1 & Mut2 vs. Mock2	1.5E-04-4.26E-02	ATP5G1,ANXA1,EEF1A1,GAPDH,KRT10,PRDX1,NPM1	7
Inflammatory Response	Mut1 vs. Mock1 & Mut1 vs. Mock2	2.36E-04-3.91E-02	CNN2,GAPDH,HMGB1,PRDX1	4
	Mut2 vs. Mock1 & Mut2 vs. Mock2	3.8E-04-4.58E-02	ANXA1,EEF1A1,GAPDH,KRT1,KRT10,PRDX1,NPM1	7
Respiratory System Development and Function	Mut1 vs. Mock1 & Mut1 vs. Mock2	3.4E-03-4E-02	DPY30,HMGB1	2
Infectious Disease	Mut1 vs. Mock1 & Mut1 vs. Mock2	1.09E-02-2.68E-02	ATP5B,CCT2,C9orf131,GAPDH,MYOF,NUP155,SUPT16H,HMGB1,PRDX1,SNRPF	10
	Mut2 vs. Mock1 & Mut2 vs. Mock2	8.25E-05-4.52E-02	ANXA1,BCLAF1,C9orf131,EEF1A1,GAPDH,MYOF,OASL,SSRP1,SUPT16H,TPT1NPM1,PRDX1,RPS16	13
Antigen Presentation	Mut1 vs. Mock1 & Mut1 vs. Mock2	3.34E-02-3.91E-02	HMGB1	1
